# Supplementary material for: Ethanolaminephosphate cytidylyltransferase is essential for survival, lipid homeostasis and stress tolerance in Leishmania major
Source: PLoS Pathog. 2023 Jul 28;19(7):e1011112. doi: 10.1371/journal.ppat.1011112 (PMC10411802; doi:10.1371/journal.ppat.1011112)
Supplement: S1 Fig — Asterisks (*): fully conserved residues; colons (:) highly similar residues; periods (.): moderately similar residues. Color code for amino acids: red-nonpolar; green-polar; blue-acidic; purple-basic. (PDF) [file ppat.1011112.s001.pdf]

```
Sc    ------------------------------------------------------------      0
Pf    MSNQFLVDTIYNHGYMRKFLSILRSVKKNDKFQYIMKLCENTNIEEDEELYKVFINELHN      60
Lm    ---------------------------------------------------------M--      1
At    -----------------------------------------------------MVWEK--      5
Hs    ------------------------------------------------------------      0

Sc    ------------------------------------------------------------      0
Pf    ISNTSSTRRKKNDSSCNESTNNTGNNKENGSNNTHFEDMKDINSSISNNSEEINEFEIDS      120
Lm    ---------PTVSSSTPSP----ASTPTVGVPPGKVW----LNADEPND----EYSLFCT      40
At    ---------EKIVGSCIVG----GAAFAVGASFLHLF----LKGELPLG----LGLGLSC      44
Hs    --------------------------------------------MIRNG----RGAAGGA      12

Sc    ---MTVNLDPDKVWIDGCFDFTHHGHAGAILQARRTVSKENGKLFCGVHTDEDIQHNKG-      56
Pf    STSTQEKTKETRIYVDGIFDLSHSGHFNAMRQAKKL----GDIVVVGINSDEDALNSKGV      176
Lm    EAIPPKVPGTVRIWVDGCFDMLHFGHANALRRARRL----GDELFVGCHSDEEVMRFKG-      95
At    PWRILRKRKPVRVYMDGCFDMMHYGHCNALRQARAL----GDQLVV--VSDEEIIANKG-      97
Hs    EQPGPGGRRAVRVWCDGCYDMVHYGHSNQLRQARAM----GDYLIVGVHTDEEIAKHKG-      67
                 ::: ** :*: * ** . : :*:      .. :.    :**:    **

Sc    TPVMNSSERYEHTRSNRWCSEVVEAAPYVTDPN----WMDKYQCQYVVHGDDITIDANGE      112
Pf    KPIYTQEERGALIAGCKWVDEVIIGTKYNVDMD----LLEKYNCDYAAHGTDLAYDKNGT      232
Lm    PPIMHAEERYEALRACKWVDHVVENYPYCTRLK----DIERFEIDYVVHGDDISVDLNGR      151
At    PPVTPLHERMTMVKAVKWVDEVISDAPYAITEDFMKKLFDEYQIDYIIHGDDPCVLPDGT      157
Hs    PPVFTQEERYKMVQAIKWVDEVVPAAPYVTTLE----TLDKYNCDFCVHGNDITLTVDGR      123
       *:    **     . :* ..*:    *    .     ::.:: ::  ** *     :*

Sc    DCYKLVKEMGRFKVVKRTYGVSTTEIIHRILTKKSLPPTHPDYYPTTQE-----------      161
Pf    CCYEEVRKFNKLKIFERSYGISTTTIINHLLQAVNNSN----YYSSSNNNNNNNNNNNNN      288
Lm    NSYQEIIDAGKFKVVKRTKGISTTDLVGRMLLCTKNHM----LKSVDEVQ----------      197
At    DAYALAKKAGRYKQIKRTEGVSSTDIVGRMLLCVRERS----ISDTHSRS----------      203
Hs    DTYEEVKQAGRYRECKRTQGVSTTDLVGRMLLVTKAHH----SSQEMSS-----------      168
        *    . .: :  :*: *:*:* :: ::*                .

Sc    ------------------------------------------L-----------------      162
Pf    NTLVNSNNNNNNNDTNSVSTNEISDINNETNYVYNTNTNSEQLDNFNKNKDNPNILEITE      348
Lm    -------------------------L--E----------------N--------------      200
At    ------------------------SLQRQ----------------F--------------      209
Hs    ----------------------------E----------------Y--------------      170

Sc    -SFYS----VA-------------------QDAVSKHCYVFQRDLDNVLVNGG--YKFDA      196
Pf    EQIYNSELGISDDNKTKVSEQQHDIDTLPKNLLNRNRCHITTSQIYQFIDNNELIKKKKN      408
Lm    -SLL--------------------------EHSPTMPCLTTSRKIVQFSNNSS---PKPG      230
At    -SHGHSSPKFEDGASS--------------AGTRVSHFLPTSRRIVQFSNGKG---PGPD      251
Hs    -REYADSFGKCPGGRN--------------PWTGVSQFLQTSQKIIQFASGKE---PQPG      212
                                                  : :.  .

Sc    EDCVYVDGDFDLFHMGDIDQLRKLKMDLHPDKKLIVGITTSD-------YSSTIMTMKER      249
Pf    KKVVYVDGSFDIFHIGHLRILENAKK-LG--DYLLVGMHSDEVVQKMKGKYFPVVSLLER      465
Lm    DRIVYVDGSFDLFHIGHIRVLQKARE-LG--DYVIAGVYEDQVVNEHKGKNYPIMSFNER      287
At    ARIIYIDGAFDLFHAGHVEILRRARE-LG--DFLLVGIHNDQTVSAKRGAHRPIMNLHER      308
Hs    ETVIYVAGAFDLFHIGHVDFLEKVHR-LAERPYIIAGLHFDQEVNHYKGKNYPIMNLHER      271
         :*: * **:** *.:  *.. :  *     ::.*:  .:           ::.: **

Sc    VLSVLSCKYVDAVIIDADATSMSQYNCEKYHIGTAVLTAA--------------------      289
Pf    TLNVLAMKVVDDVVIGAPWVITE-SFIKRFHIDVVVRGTIVDY-IYSNNEIDPYDIPKKL      523
Lm    VLGVLSCRYVDEVVMGVPFDVSK-DVIDGLHINVVVGDKFSDL-VVEEGGSTRYEVPKAM      345
At    SLSVLACRYVDEVIIGAPWEVSR-DTITTFDISLVVHGTVAESDDFRKEEDNPYSVPISM      367
Hs    TLSVLACRYVSEVVIGAPYAVTA-ELLSHFKVDLVCHGKTEIIPD--RDGSDPYQEPKRR      328
       *.**: : *. *::..             .:. .

Sc    GKF-----SEYLTKELIVKRVESQREVYIARNQKKGMSI---------------------      323
Pf    NIYQELSSESNITTYEIIQRIEKNKKY-LMRNMSKRNKKEESIWETSNTYAINN------      576
Lm    GIYHEVDSGCILSTDSLIDRVVENRLDFLKRQAEKRI-KDTKSQEIKPDEYRK-LREAS-      402
At    GIFQVLDSPLDITTSTIIRRIVANHEAYQKRNAKKEA-SEKKYYEQKSFVSGD-------      419
Hs    GIFRQIDSGSNLTTDLIVQRIITNRLEYEARNQKKEA-KELAFLEAARQQAAQPLGERDG      387
      . :        ::.  :: *:  ::     *: .*

Sc    -- 323
Pf    -- 576
Lm    -- 402
At    -- 419
Hs    DF 389
```
