# Supplementary figures and images for: Ethanolaminephosphate cytidylyltransferase is essential for survival, lipid homeostasis and stress tolerance in Leishmania major
Source: PLoS Pathog. 2023 Jul 28;19(7):e1011112. doi: 10.1371/journal.ppat.1011112 (PMC10411802; doi:10.1371/journal.ppat.1011112)

**A**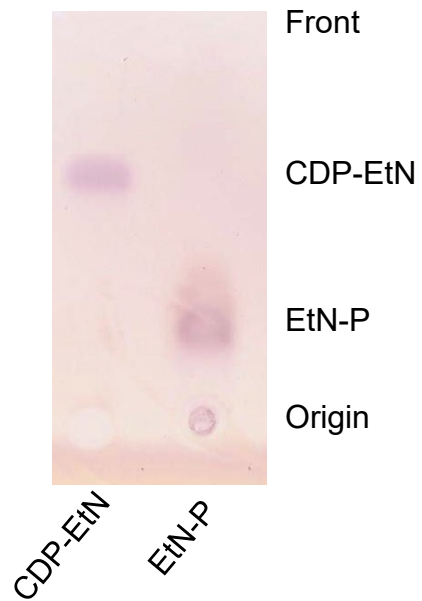**B**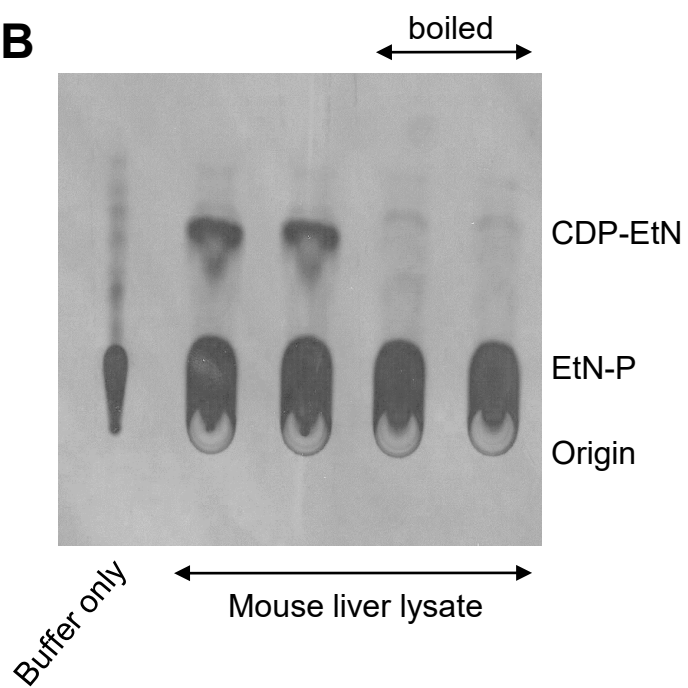

Supplement: S2 Fig — (A) EtN-P or CDP-EtN (40 nmol each) are resolved by TLC as described in Materials and Methods. The plate was dried and sprayed with 0.2% ninhydrin to show the positions of EtN-P and CDP-EtN. (B) Mouse liver lysates (boiled and not boiled, two repeats each) were incubated with [14C]-EtN-P at room temperature, followed by TLC analysis and signals were detected by autoradiography. (PDF) [file ppat.1011112.s002.pdf]

Fig. S3

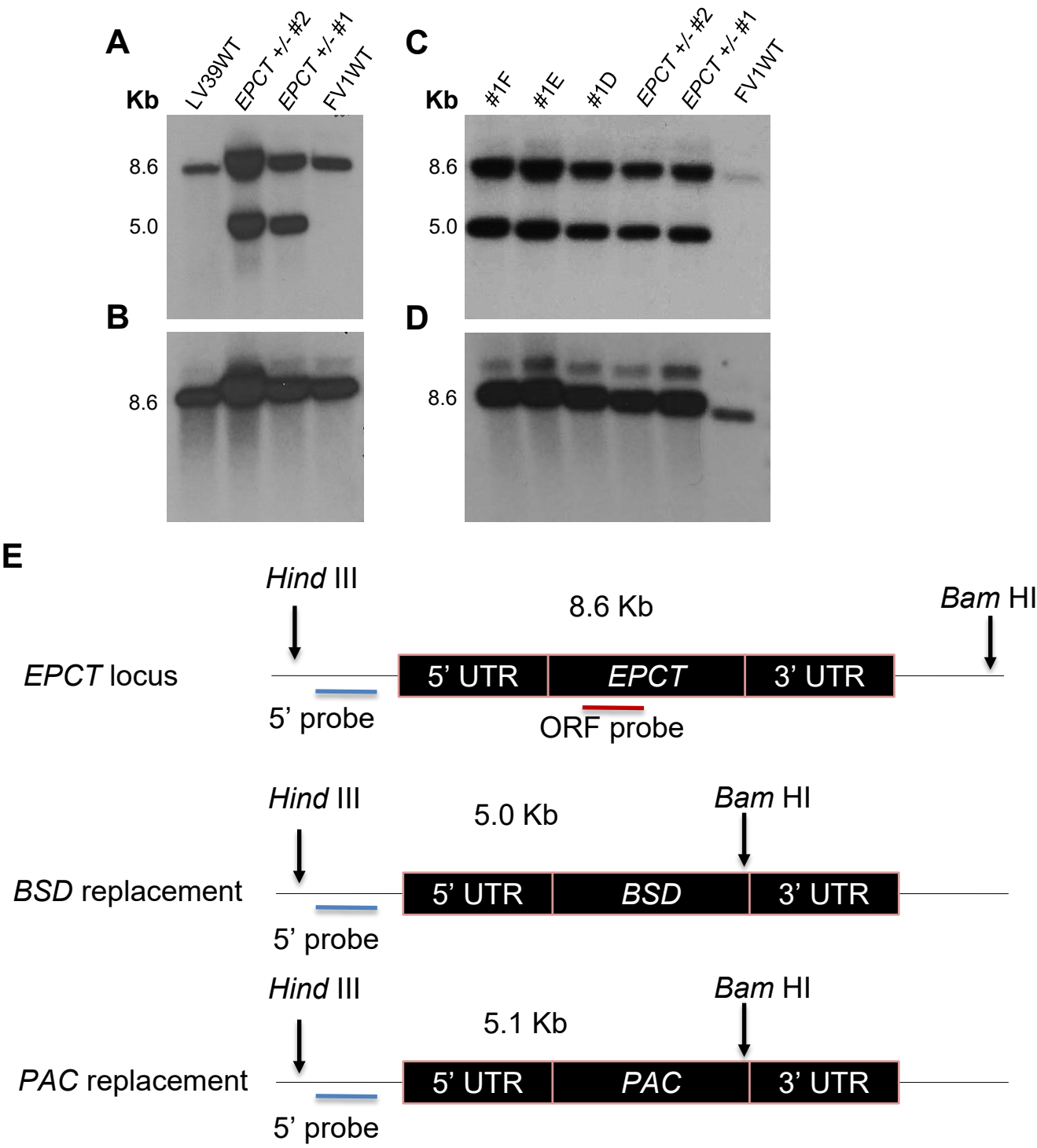

Supplement: S3 Fig — Genomic DNA samples from L. major FV1 WT, LV39 WT, EPCT+/- (#1 and #2), and putative epct¯ (#1D, #1E, and #1F) parasites were digested by Hind III + Bam HI followed by Southern blot analyses using radiolabeled probes for an upstream flanking sequence (5’ probe: A and C) and the open reading frame of EPCT (ORF probe: B and D). The approximate recognition sites of Hind III and Bam HI and expected DNA fragment sizes are indicated in E. (PDF) [file ppat.1011112.s003.pdf]

Fig. S4

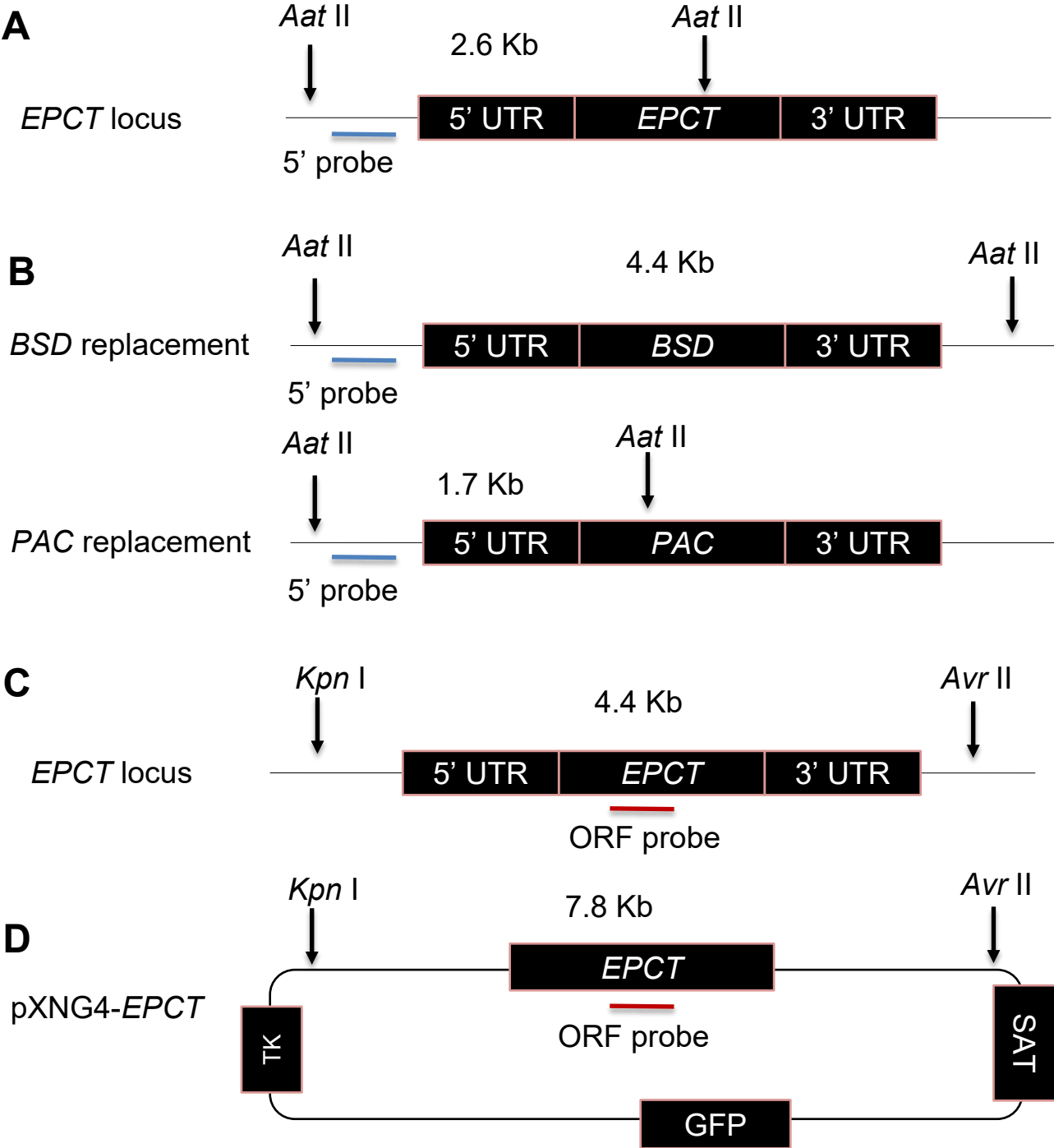

Supplement: S4 Fig — Expected DNA fragment sizes for using the 5’ probe (A-B) or ORF probe of EPCT (C-D) are indicated. TK: thymidine kinase, GFP: green fluorescent protein, SAT: nourseothricin resistance gene. (PDF) [file ppat.1011112.s004.pdf]

Fig. S5

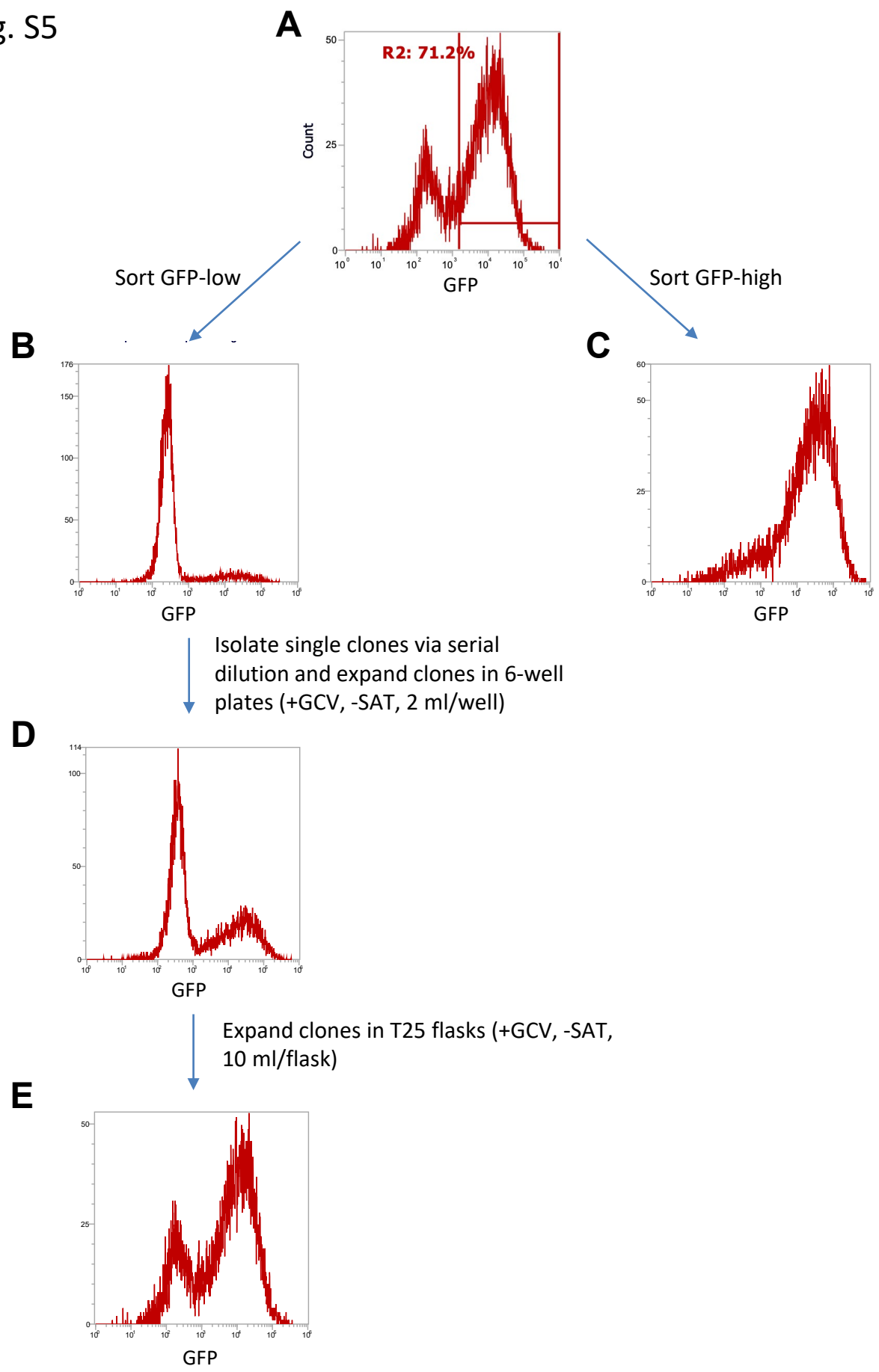

Supplement: S5 Fig — (A) Epct¯+pXNG4-EPCT promastigotes were cultivated in the presence of GCV and absence of SAT for 14 passages as indicated in Fig 4D. GFP-low population (B) was separated from GFP-high population (C) by FACS. Single clones were then isolated from the GFP-low population (B) via serial dilution followed by two consecutive rounds of expansion in the presence of GCV and absence of SAT (D and E represent the first and second round of expansion respectively). GFP expression levels in A-E were analyzed by flow cytometry. (PDF) [file ppat.1011112.s005.pdf]

Fig. S7

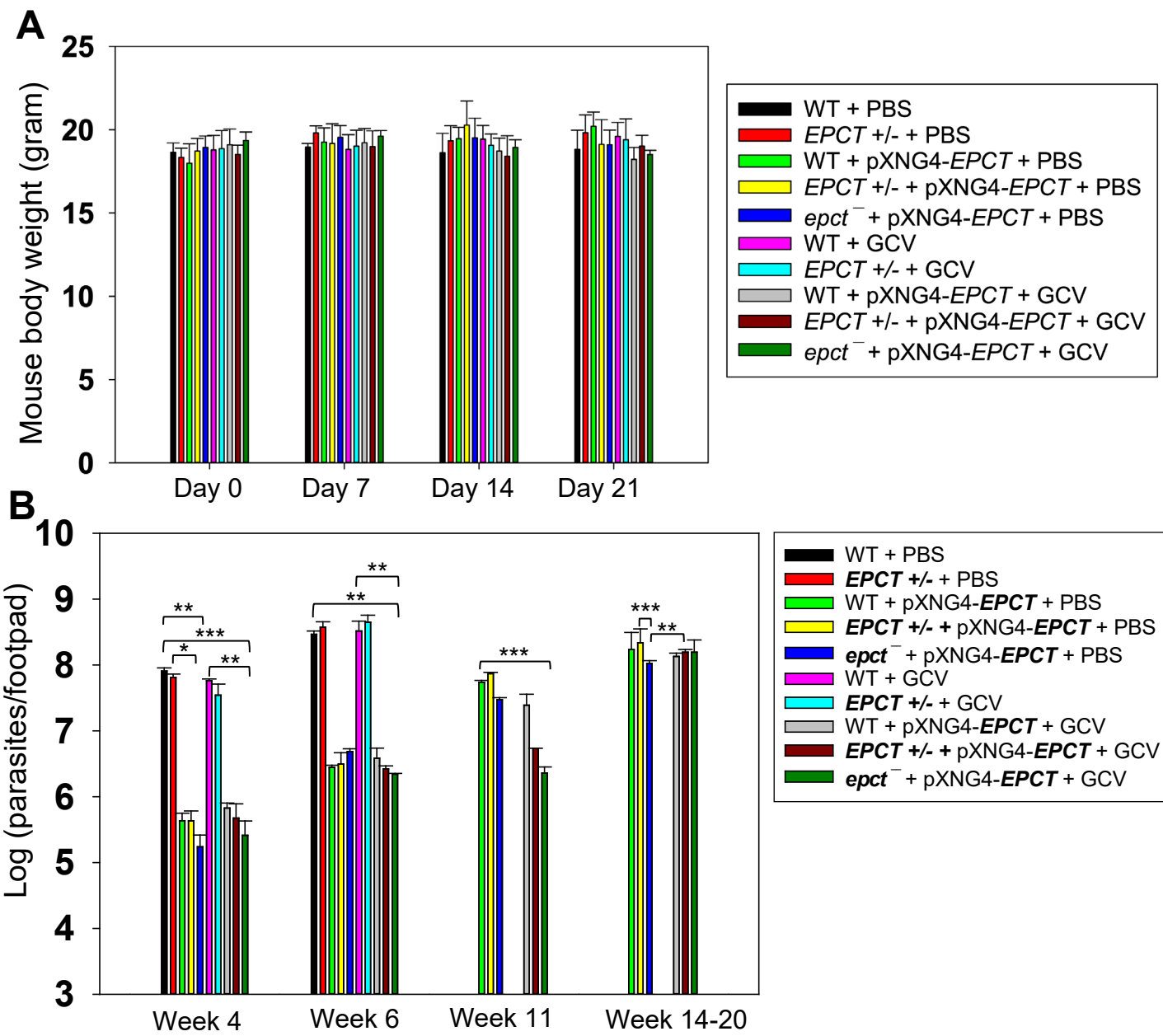

Supplement: S7 Fig — Following footpad infection, mice were treated with GCV or PBS and euthanized at the indicated timepoints. (A) Mouse body weights were measured at day 0–21 post infection. (B) Genomic DNA samples were prepared from lesion-derived amastigotes and parasite loads were determined by qPCR using primers targeting the L. major 28S rDNA gene (*: p < 0.05, **: p < 0.01, ***: p < 0.001). (PDF) [file ppat.1011112.s007.pdf]

Fig. S8

**A**

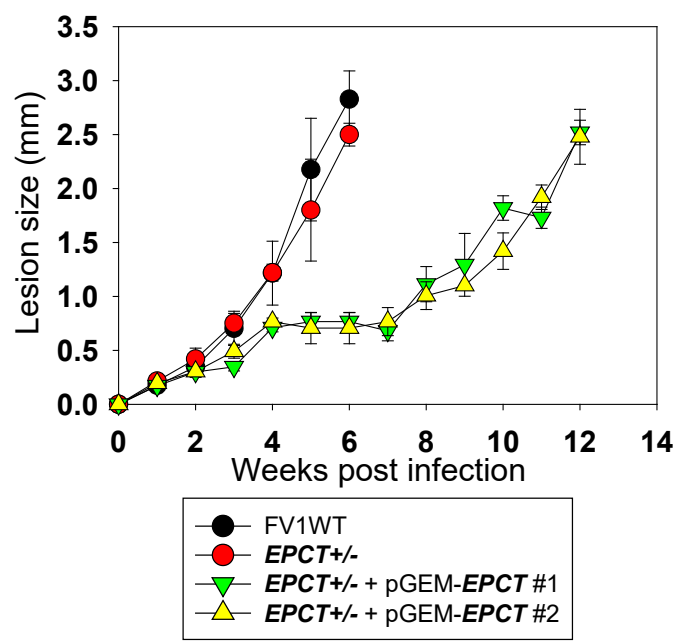

**B**

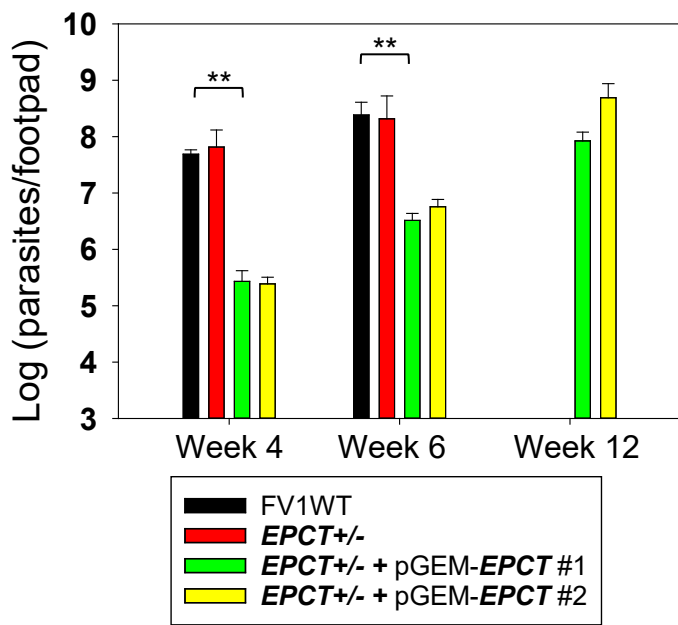

Supplement: S8 Fig — Stationary phase promastigotes were injected into the footpad of BALB/c mice as described in Materials and Methods. Footpad lesion sizes were measured using a Vernier caliper (A) and parasite loads were determined by qPCR (B). **: p < 0.01. (PDF) [file ppat.1011112.s008.pdf]

Fig. S9

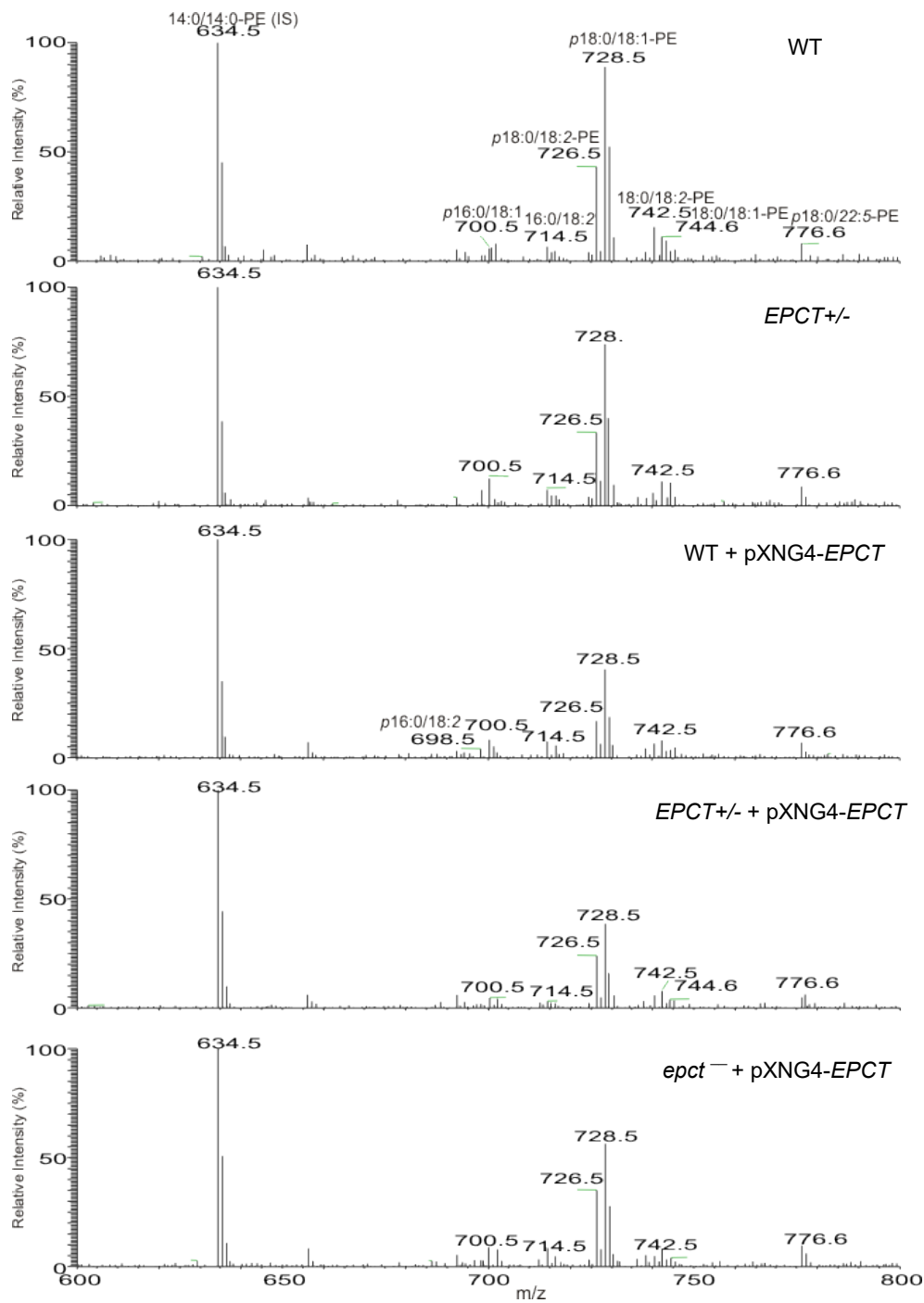

Supplement: S9 Fig — Total lipids were extracted from log phase promastigotes and analyzed by ESI-MS in the negative ion mode as described in Materials and Methods. The 14:0/14:0-PE (m/z 634.5) was added as an internal standard. Representative tandem mass spectra obtained from precursor ion scan of m/z 196 specifically monitoring PME and diacyl-PE species were shown for WT, EPCT+/-, WT + pXNG4-EPCT, EPCT+/- + pXNG4-EPCT, and epct¯ + pXNG4-EPCT. Major PME species such as p18:0/18:2-PE (m/z 726.5) and p18:0/18:1-PE (m/z 728.5) were indicated. (PDF) [file ppat.1011112.s009.pdf]

Fig. S10

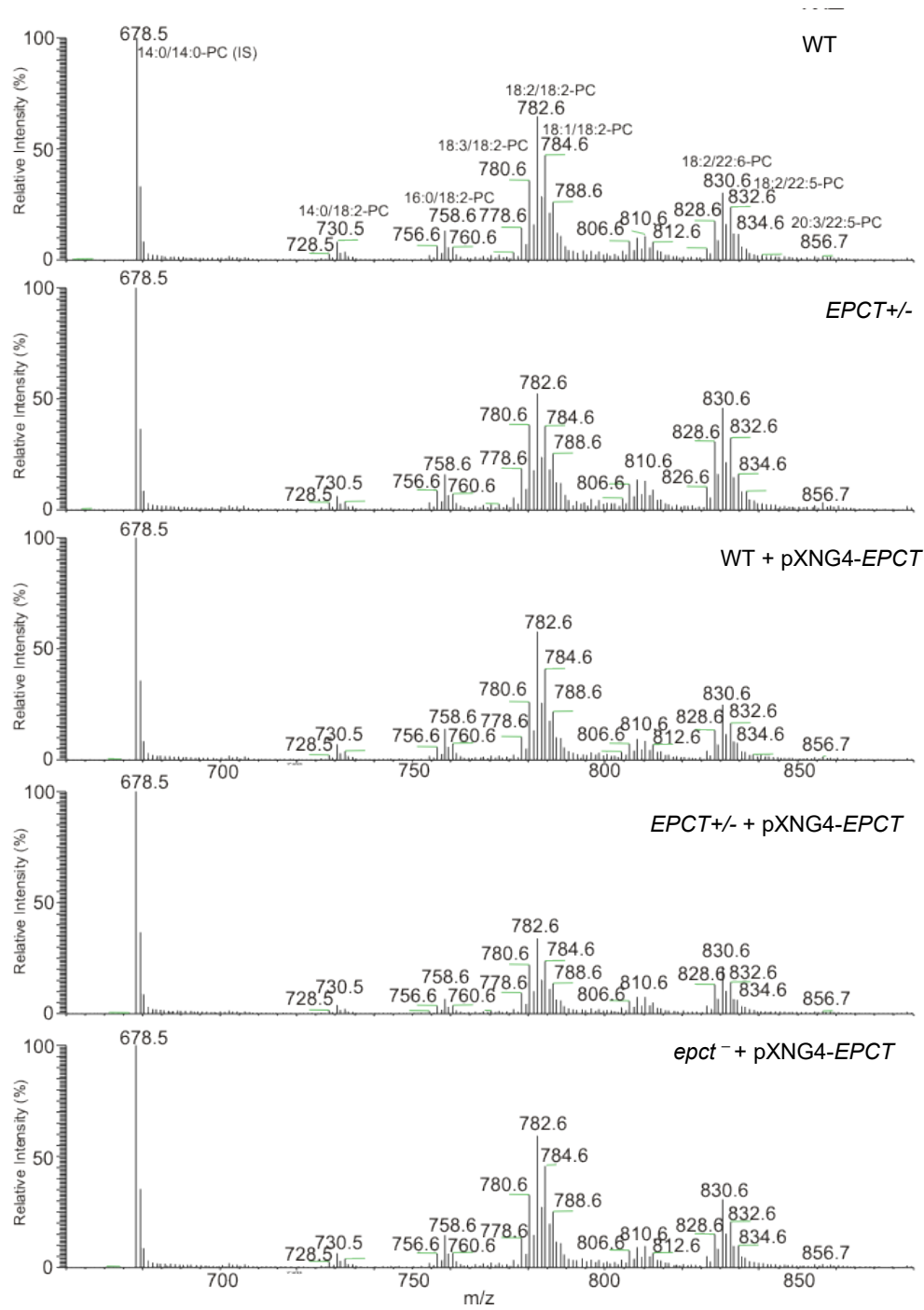

Supplement: S10 Fig — Total lipids were extracted from log phase promastigotes and analyzed by ESI-MS in the positive ion mode as described in Materials and Methods. The 14:0/14:0-PC (m/z 678.5) was added as an internal standard. Representative tandem mass spectra obtained from precursor ion scan of m/z 184 specifically monitoring PC and sphingomyelin species were shown for WT, EPCT+/-, WT + pXNG4-EPCT, EPCT+/- + pXNG4-EPCT, and epct¯ + pXNG4-EPCT. Major PC species such as 18:2/18:2-PC (m/z 782.6) and 18:2/22:6-PC (m/z 830.6) were illustrated. (PDF) [file ppat.1011112.s010.pdf]

Fig. S11

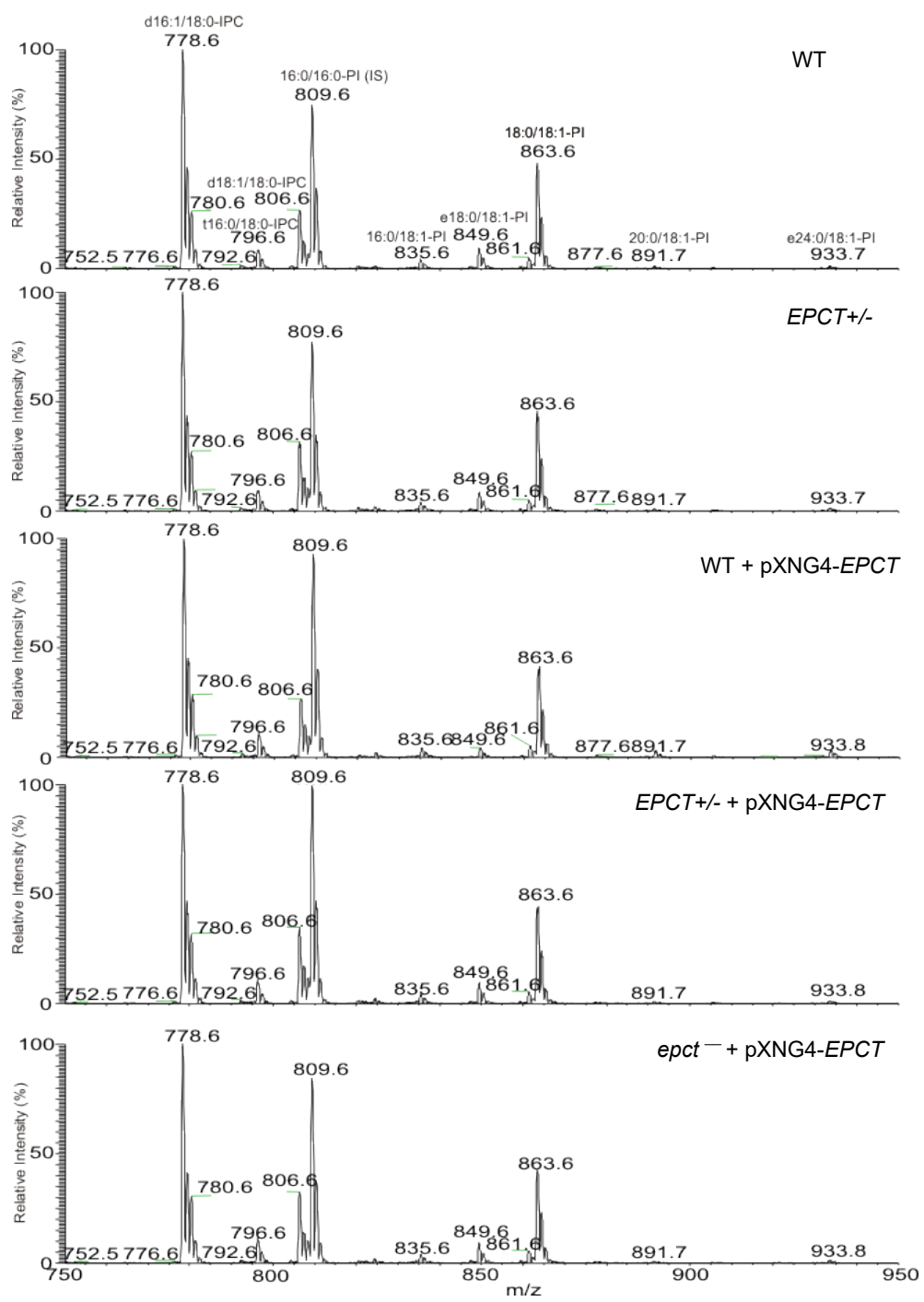

Supplement: S11 Fig — Total lipids were extracted from log phase promastigotes and analyzed by ESI-MS in the negative ion mode as described in Materials and Methods. The 16:0/16:0-PI (m/z: 809.6) was added as an internal standard. Representative tandem mass spectra obtained from precursor ion scan of m/z 241 (specific for PI) were shown for WT, EPCT+/-, WT + pXNG4-EPCT, EPCT+/- + pXNG4-EPCT, and epct¯ + pXNG4-EPCT. Major PI species were indicated. (PDF) [file ppat.1011112.s011.pdf]

Fig. S12

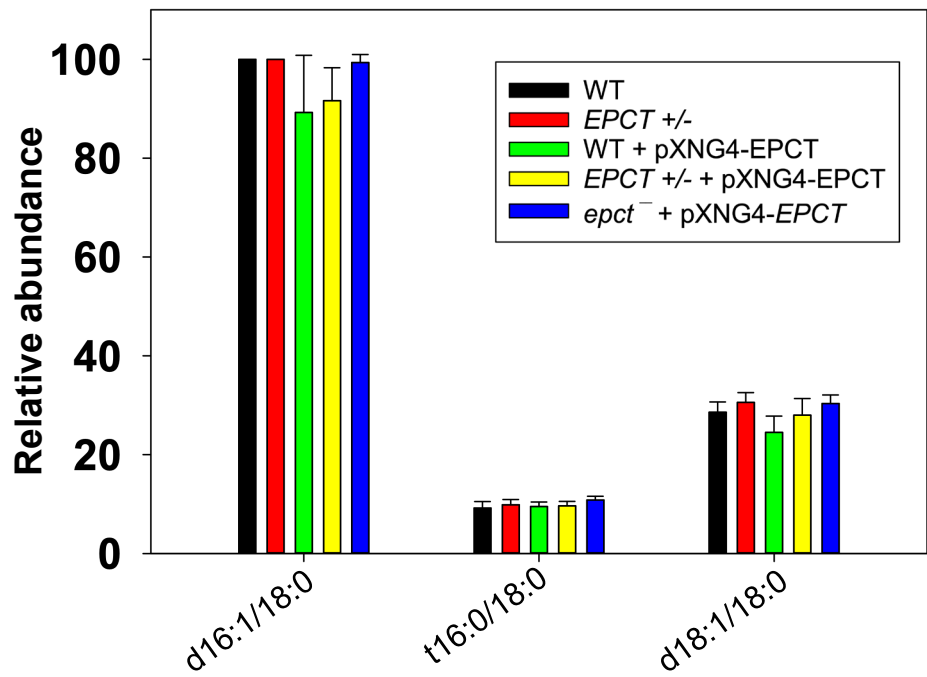

Supplement: S12 Fig — Total lipids were extracted from stationary phase promastigotes and analyzed by ESI/MS in the negative ion mode using both total ion current scan and precursor ion scan of m/z 241. Error bars represent standard deviations from 4 independent experiments. (PDF) [file ppat.1011112.s012.pdf]

Fig. S13

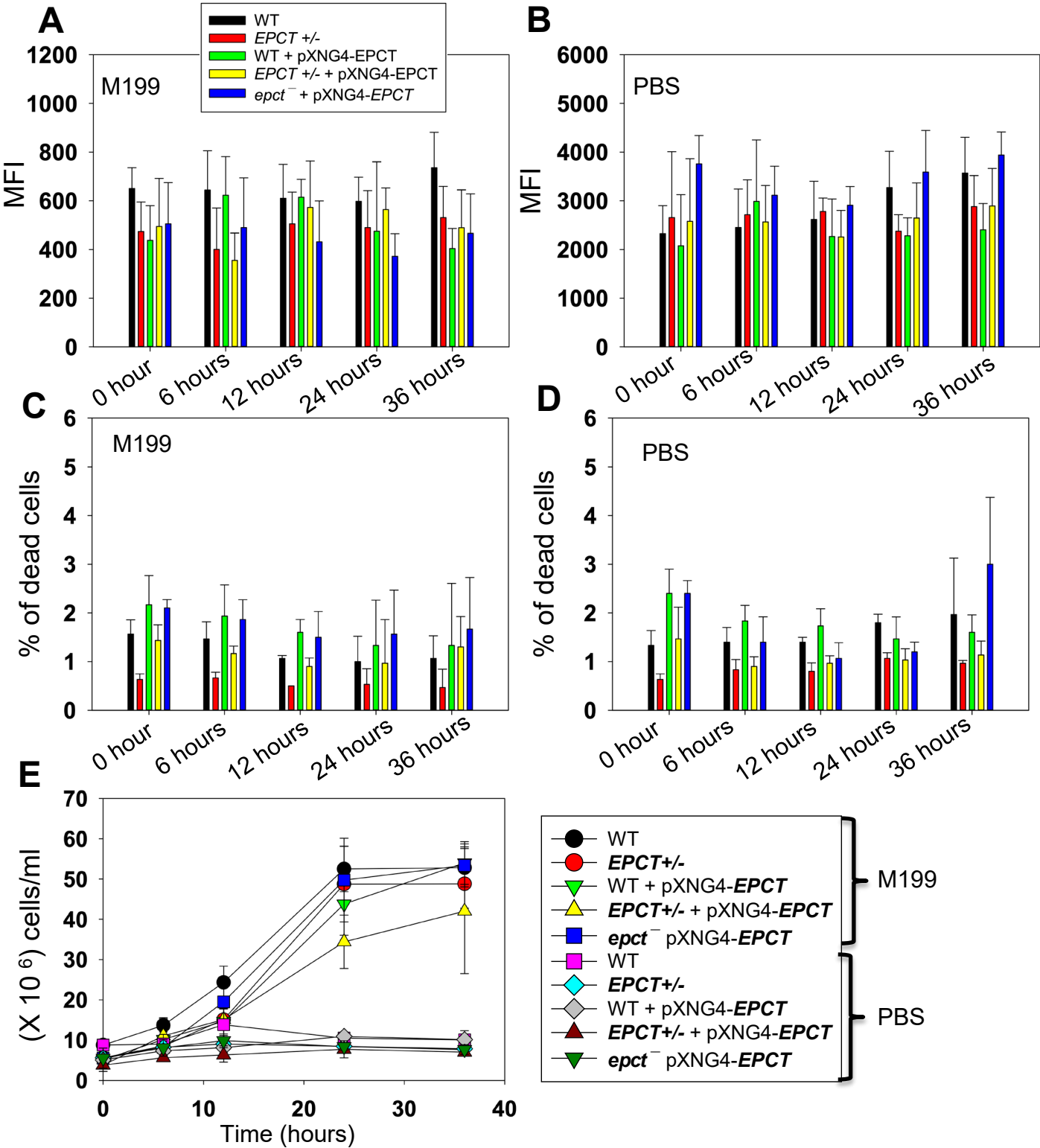

Supplement: S13 Fig — Log phase promastigotes were cultivated in complete M199 medium (A, C) or transferred to PBS (B, D) and labeled with MitoSox Red for 25 min at ambient temperature. Mean fluorescence intensity (MFI) for MitoSox Red (A, B) and percentages of dead cells (C, D) were determined by flow cytometry at the indicated timepoints. Cell growth rates in M199 or PBS were determined by hemocytometer counting (E). Error bars represent standard deviations from three independent experiments. (PDF) [file ppat.1011112.s013.pdf]
