# Supplementary material for: Ethanolaminephosphate cytidylyltransferase is essential for survival, lipid homeostasis and stress tolerance in Leishmania major
Source: PLoS Pathog. 2023 Jul 28;19(7):e1011112. doi: 10.1371/journal.ppat.1011112 (PMC10411802; doi:10.1371/journal.ppat.1011112)

Fig. S6

A

Cell Type	*epct*⁻ + pXNG4-*EPCT* + SAT (pool)	*epct*⁻ + pXNG4-*EPCT* + GCV (GFP low clone)	*EPCT*+/- + pXNG4-*EPCT* GCV (GFP low clone)
pXNG4-*EPCT* copies/cell	25 ± 2.1	8.2 ± 1.2	0.0031 ± 0.0020

B

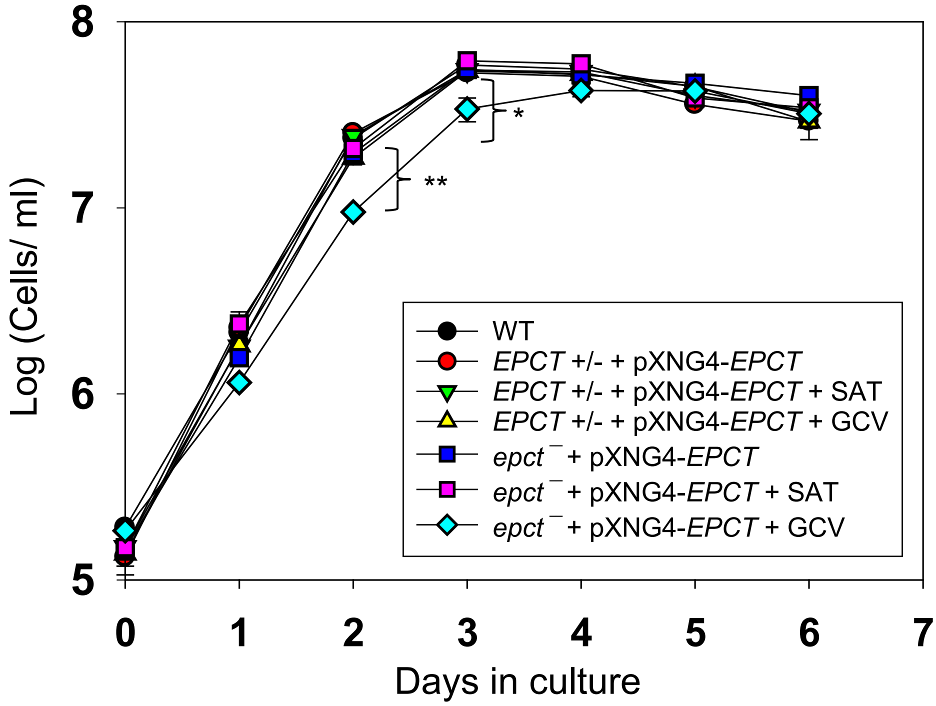

Supplement: S6 Fig — (A) EPCT+/- + pXNG4-EPCT and epct¯ + pXNG4-EPCT promastigotes were cultivated in the presence of SAT or GCV for 14 passages (as pools) and individual clones were isolated via FACS followed by serial dilution as described in Materials and Methods. Plasmid copy number numbers (average ± SDs) were determined by qPCR. (B) Promastigotes were cultivated at 27°C in complete M199 media and culture densities were determined daily using a hemocytometer. Error bars indicate standard deviations from three biological repeats (*: p < 0.05, **: p < 0.01). (PDF) [file ppat.1011112.s006.pdf]
