# Supplementary material for: Ethanolaminephosphate cytidylyltransferase is essential for survival, lipid homeostasis and stress tolerance in Leishmania major
Source: PLoS Pathog. 2023 Jul 28;19(7):e1011112. doi: 10.1371/journal.ppat.1011112 (PMC10411802; doi:10.1371/journal.ppat.1011112)
Supplement: S1 Table — Sequences in lowercase represent restriction enzyme sites. (PDF) [file ppat.1011112.s014.pdf]

Supplemental Table S1.

Primer	Name	Sequence
#52	5’ UTR EPCT forward	CATGACgaattcGACCAGCAACGTGAGGGAC
#12	5’ UTR EPCT reverse	TCAGACACTAGTGATCATGGATCCGGTGGCGGCAGAAGTGGAAG
#13	3’ UTR EPCT forward	TCAGTAggatccCGTAGTGGGCTGGCGGGGAG
#14	3’ UTR EPCT reverse	GATCATaagcttCAAATAAAGAGAGTCAGTG
#9	EPCT ORF forward	GATCAGggatccACCATGCCCACCGTTTCTTCG
#10	EPCT ORF reverse	GACTACggatccCTAGCTTGCCTCCCGTAATTTG
#73	EPCT 5’ UTR Southern forward	AGCACTTGCTCCAAGCGAAGAG
#74	EPCT 5’ UTR Southern reverse	GAACAAGAAGCCGTACTTCACAG
#15	EPCT ORF Southern forward	GCCGAGGAGCGCTATGAGGC
#16	EPCT ORF Southern reverse	GAGAACTTGTCGCCTACAAC
#699	28S rRNA qPCR forward	AAGATGGACCGGCCTCTAGT
#700	28S rRNA qPCR reverse	ATCCTTCCCCGCTCCAGTAT
#703	pXNG4 qPCR forward	CCCGACAACCACTACCTGAG
#704	pXNG4 qPCR reverse	GTCCATGCCGAGAGTGATCC
#842	EPCT ORF RT forward	CATGAGCTTCAACGAGCGTG
#843	EPCT ORF RT reverse	GCCGTCAATCACATCCTTGC
