# Supplementary material for: Ethanolaminephosphate cytidylyltransferase is essential for survival, lipid homeostasis and stress tolerance in Leishmania major
Source: PLoS Pathog. 2023 Jul 28;19(7):e1011112. doi: 10.1371/journal.ppat.1011112 (PMC10411802; doi:10.1371/journal.ppat.1011112)
Supplement: Attachment — Submitted filename: Response to review 05-2023.docx [file ppat.1011112.s015.docx]

We thank the reviewers for their constructive comments and insightful discussion. Our point-by-point response is listed below in red.

**Part I - Summary**
Please use this section to discuss strengths/weaknesses of study, novelty/significance, general execution and scholarship.

Reviewer #1: This is a solid piece of work for the most part. There are some minor issues that need to be addressed.
In terms of novelty of the work, I was surprised by the lack of acknowledgement that this gene has been published on in very closely related T.brucei and there is no reference to this work.

We have now mentioned the *Tb* EPCT studies in the Introduction (line 136-139).

There are far too many figures in the main manuscript and this should be reduced to the normal 8.

We have consolidated the three lipidomic figures into one (Fig. 7). Representative tandem mass spectra are now shown in Supplemental Fig. S9-S11.

Reviewer #2: This manuscript describes an intriguing analysis of the second enzyme in the ethanolamine branch of the “Kennedy” pathway, ethanolamine phosphate cytidyltransferase (EPCT) within Leishmania major promastigote and amastigote lifecycle stages. The key takeaways are that
• the EPCT gene appears to be essential in both the promastigote and amastigote lifecycle stages and deletion of both EPCT chromosomal copies in promastigotes could only be achieved in the presence of a complementing episome.
• the episomal overexpression of EPCT caused a significant growth detect in both epct-/- promastigotes, as well as in WT, EPCT+/-, and epct-/- lesion derived amastigotes, and rendered these parasites more susceptible to nutrient starvation. Likewise, epct-/- promastigotes expressing EPCT from an episome were less tolerating of changes in pH and temperature.
• episomal overexpression of EPCT leads to dysfunction in the regulation of the “Kennedy” pathway and the synthesis of its downstream products.
• disruption of the ethanolamine metabolism by either deletion of one or both EPCT alleles or overexpression of EPCT from an episome affects the synthesis and surface expression of the GPI-anchored protein, GP63.
Overall, the systematic approach described in this manuscript makes a compelling case for EPCT as an important new drug target for future exploration in Leishmania. However, while the majority of the experiments described in the manuscript were sophisticated and well-executed, and the experimental outcomes convincing, there were a few areas where the manuscript could be improved, which I have detailed below.

Reviewer #3: In this study, the authors investigate the functional importance of an enzyme involved in lipid synthesis, Ethanolaminephosphate cytidyltransferase (EPCT), in Leishmania donovani promastigotes and amastigotes. This enzyme catalyzes the condensation of CTP with Ethanolamine phosphate to generate CDP-Ethanolamine, which is then used to generate both major sub-classes of ethanolamine phospholipids: plasmenylethanolamines (PME; major type in Leishmania) and diacyl-phosphatidylethanolamines (diacyl-PE). The authors generated heterozygote and homozygote null EPCT mutants complemented by an episomal EPCT copy that could be controlled by either positive or negative antibiotic selection. The authors then examined several phenotypic aspects of the mutants: (1) in vitro and in vivo growth of the mutants and their ability to lose the episomal copy; (2) EPCT activity, localization, and expression; (3) lipidomic changes; (4) downstream effects on lipid-anchored cell surface markers; and (5) impact on stress response.

One major finding of the work is that EPCT is essential in both promastigotes and amastigotes, based on the inability to generate homozygous nulls unless complemented with an episomal copy that cannot be lost, even under negative selection. A second major finding is that over-expression of EPCT is deleterious to the parasite, showing negative impacts on promastigote growth in culture and amastigote growth in mice (less virulent), reduction in PME and inositolphosphorylcholine (IPC) lipid species, reduction in GP63 surface expression, and impaired stress tolerance (starvation, acid, and heat). These major findings are clear and well-supported by the data and included controls.

One major strength of these findings is that they highlight the indispensable importance of EPCT to the parasite, even in the mammalian amastigote stage. This is in contrast to current thinking, based on results with other lipid synthesis mutants, that the amastigotes are less dependent upon their own lipid synthesis because they grow more slowly and can instead rely upon uptake of host lipids and lipid precursors. The results in this manuscript show that EPCT activity is still needed in the amastigote stage, implying that host resources or their acquisition is insufficient. A second strength is the very clear implication that too much EPCT activity is broadly deleterious for Leishmania, implying that a balanced tuning of lipid synthesis pathways is essential.

The manuscript does have some moderate weaknesses in its current form, which are summarized here and detailed below. The methods used for the studies shown in Fig. 4 and Fig. S5 are not described in sufficient detail for this reviewer to evaluate the data and conclusions. There is an inconsistency in the methods vs. the results with the generation of the null mutants. The potentially mixed effects of toxicity from ganciclovir treatment with toxicity from EPCT over-expression are not adequately addressed. Some speculation of effect on GP63 expression is needed. Finally, the results of the EPCT RNA expression data are confusing and not adequately addressed by the authors.

We have provided more details on the sorting experiments in Fig. 4 and Supplemental Fig. S5. We have corrected the inconsistency in the description of *EPCT* mutant generation and provided more discussion on GCV treatment, GP63 abundance and EPCT mRNA as described below.

Overall, the manuscript has many strengths and represents a generally careful and thorough evaluation of the functional importance of EPCT in Leishmania. The key observations are well-supported and reveal that there is something special about the PE synthesis pathway, and more specifically, EPCT, which is needed in amastigote form and thus, constitutes an exciting new candidate drug target. So the work should be of interest to those working on drug development in eukaryotic pathogens. This research also will be of great interest to others focused more generally in lipid synthesis in kinetoplastids and other protozoan pathogens.

**Part II – Major Issues: Key Experiments Required for Acceptance**
Please use this section to detail the key new experiments or modifications of existing experiments that should be absolutely required to validate study conclusions.

Generally, there should be no more than 3 such required experiments or major modifications for a "Major Revision" recommendation. If more than 3 experiments are necessary to validate the study conclusions, then you are encouraged to recommend "Reject".

Reviewer #1: There are four experimental issues that should be addressed.
1) in fig 2Athe authors use over expressing EPCT in the Leish background to show for activity. This is done by the level of the upper "CDP-Etn" band. Granted we see a more intense ban in the OE cell lines, but there is presence of this band in the buffer only (which is very concerning" and also the boiled (so dentures protein) controls, why would this be so. I would like to suggest that may be the higher band is not CDP-EthN, but may be Etn that has no P , in other words it has been dephosphorylkated. This could arise by the radioactive Etn-P partially degraded or phosphatase activity present, which there will be lots of in the lysate. Show me on a TLC where EthN runs rot be certain.

We agree that the residue amount of CDP-EtN-like material seen with buffer only and boiled lysates were likely due to impurities in [^14^C]-EtN-P or spontaneous hydrolysis. However, we believe most signals from EPCT overexpressors were representative of [^14^C]-CDP-EtN because the Rf value on TLC was consistent with our control using pure, non-radiolabeled CDP-EtN (Supplemental Fig. S2A) and the Rf value of CDP-EtN from literature (PMID: 15147238). Quantified analyses from four repeats indicate only WT+EPCT and WT+GFP-EPCT have clear EPCT activities (Fig. 2B).

Fig 2B shows the anti-GFP protein bands which show several in the middle lane... thus when this is translated to the IF, then not only is the full length fusion showing up but also the truncated versions.. all of which are being visualised in the IF images. Localisations in cells normally requires a sub-cellular fractionation and IF, so the authors need to do a simple sub-cellular fraction western, then at least then they can be certain that the full length GFP +ve band is in the cytsol. also why do DAPI staining?

We repeated the anti-GFP western blot using log phase promastigotes and a lower temperature for denaturation (65 °C instead of 95 °C in the previous western blot). Results were much cleaner as GFP-EPCT was detected at the predicted 73 KD size without significant degradation (Fig. 2C). We also repeated the fluorescence microscopy experiment with DNA staining and results confirmed the cytoplasmic localization of GFP-EPCT (Fig. 2D).

The authors clearly show that the levels of certain lipid species are decreased both in the cells over expressing or under expressing the EPCT. So the authors look at PE and PC, but also PI and IPC species, however the decrease in lipid content by these species, must be compensated for by another lid, which the authors have not shown, please show the full survey scans.. what else is going on? The cells obviously divide at a reasonable rate, so is their morphology or cell volume changed.. we do see any images of these modified cell. In the T.brucei work clear shows the cell volume has decreased, as well as the mitochondria... which the authors here do not even consider investigate with mitotracker, despite PE being so important for the Mito.

The reviewer raised a good point as whether the decrease in one lipid class is compensated by increase in other lipids. Since PE (PME + diacyl PE) represents ~10% of total cellular lipids, the PME reduction in EPCT overexpressors (Fig. 7A and Supplemental Fig. S9) would account for 3-4% loss of total lipids. No other significant changes were observed in the total ion current analyses of phospholipids. Nonetheless, it is possible that EPCT over- or under-expression may change the levels of neutral lipids. These points are now mentioned in line 377-380.

We did not observe any significant change in cell shape/size or growth in EPCT mutants under normal promastigote culture conditions (Figs. 2D, 3E and 8A), although EPCT overexpression did cause stress response defects and reduced virulence (Fig. 5 and Fig. 9). With Mitosox staining, we did not find any significant difference in mitochondrial ROS production, suggesting that PE synthesis in the mitochondria is not affected (Supplemental Fig. S13).

Reviewer #2: Major issues to be addressed:
• It’s unclear how the levels of GP63 were quantified for the different cell lines described? It appears that the values were derived from the immunofluorescence images captured. I have reservations about the accuracy of this approach, since I imagine it can be affected by any number of things including changes in the sample processing, gain and exposure settings, differences in the focal plane. At the very least, it would have been helpful to provide some indication for the number of cells enumerated and included another fluorescence marker (e.g., anti-tubulin) as a normalization control for the image analysis. A better and more standard approach would have been to include a quantitative western analysis using the anti-GP63 antisera.

We could not use western blot to quantify GP63 like we did for LPG because the anti-GP63 antibody (#96/126, Abcam Inc) only works on native GP63 but not denatured protein. In fact, all commercial anti-GP63 antibodies are derived from the same monoclonal hybridoma. In the past, we were able to request other anti-GP63 antibodies such as the mAB 235 or rabbit polyclonal antibody from other research groups for western blot. However, we ran out of those antibodies and were unable to acquire more. To ensure the accuracy of quantitation by microscopy, all cells were processed with the exact same settings in staining, image acquisition (exposure and focus), and image enhancement. In addition, we performed new immunofluorescence experiments and measured the cellular level of GP63 by dividing the integrated density of GP63 fluorescence minus background with the integrated density of Hoechst (DNA staining) minus background. For each cell type, 200 cells were analyzed, and the average of GP63/Hoechst was determined and normalized to WT (as 1.0). This procedure is described in line 492-499. Results from the new experiment (Fig. 8A and C) are highly consistent with the previous experiment which did not use DNA stain as an internal control.

Reviewer #3: Specific comments:

1. Fig 4 (Panels C-F) and Fig. S5 (Panel A) – Post-Sorting Recovery of High-GFP population. The methods and interpretation of this experiment are unclear. The GFP-low population in D (epct– + pXNG4-EPCT + GCV) was isolated by cell sorting then clones isolated by serial dilution. The population immediately after isolation is not shown, so quality of sorting is not able to be evaluated. Also it is not clear how the cells were propagated after sorting & cloning. During clone outgrowth, was GCV absent, was SAT present, or were all antibiotics omitted? Not clear how conclusion on Line 208 “GFP-low cells were only viable in the presence of GFP-high cells” is reached? What is this based on? Cannot follow that conclusion’s logic here. To me, this just seems like the recovery of the “high-GFP” population just reflects removal of GCV-negative selection on the episomal copy, and subsequent expansion of episomal copy number. qPCR of episomal copy number was performed (Fig. S5A), but it is not clear what time point after clonal isolation and outgrowth and in what culturing conditions (presence of antibiotics) were the samples taken for qPCR analysis. Was this immediately after cell sorting out the low GFP population?

We have now provided more details for the sorting experiment (line 512-517 and 702-708, Supplemental Fig. S5). The GFP-low and GFP-high populations immediately after FACS were analyzed to confirm the quality of sorting (Supplemental Fig. S5B-C). Individual clones from GFP-low population were expanded in the presence of GCV and absence of SAT for two consecutive passages and results showed rapid increase in GFP-high percentages and plasmid copy numbers (Supplemental Fig. S5D-E and Fig. 6A). These data suggest that GFP-low cells of *epct¯*+ pXNG4-EPCT would increase the episome level during proliferation after they were separated from GFP-high cells. Thus, these cells can only survive and proliferate in the presence of GFP-high cells (line 210-218 and 350-355).

2. Ganciclovir (GCV) treatment is toxic to cells expressing Thymidylate Kinase (TK) on the episome, which serves as a negative selection for the episomal EPCT copy. However, cells that cannot lose the episomal EPCT are potentially suffering from TWO effects that are happening at the same time: toxicity from GCV and toxicity from EPCT over-expression. I would like to see a little more discussion of this, and can your data shed any light on the relative contribution of GCV toxicity vs. EPCT over-expression might be having to the impaired growth you’re seeing in Fig. S5B and Fig. 5. I think there are some clues in your data that GCV toxicity might be contributing more to impaired growth in vivo in amastigotes than in Promastigotes, cross-correlating episome copy number vs. EPCT mRNA levels.

*Epct¯*+ pXNG4-EPCT amastigotes retained 12-24 plasmids per cell throughout the course of mouse infection, despite the cytotoxic effects from GCV and EPCT overexpression (Fig. 5C). We also detected elevated *EPCT* transcript from *epct¯*+ pXNG4-EPCT amastigotes isolated from GCV-treated mice (Fig. 6C). Based on these findings, we postulate that the need for EPCT outweighs the potential negative impacts from GCV and EPCT overexpression for *L. major* during the mammalian stage. It is important to remember that GCV treatment of mice only lasted for the first two weeks post infection, and its effect likely wore down in the later stage of infection. In addition, plasmid copy number and EPCT transcript level may not fully reflect the EPCT protein level (line 404-411).

3. In addition to the point above, I’d like to see some attempt in the Discussion to consider the phenotypes in light of the episome copy number and EPCT mRNA data. Particularly as the Fig. 6C EPCT mRNA expression data is surprising, showing that GCV treatment increased EPCT mRNA levels in two of the EPCT over-expressing lines, when I would have expected it to decrease due to negative selection on the episome. The data is all a little puzzling as the in vivo lesion progression, the EPCT mRNA, and the episomal copy numbers don’t correlate very well.

We have elaborated more on these results (line 280-288). For *EPCT+/-* +pXNG4-EPCT and *epct¯*+ pXNG4-EPCT amastigotes (week 14 post infection), EPCT transcript levels correlated with plasmid copy numbers (Fig. 5C and Fig. 6C). The fact that GCV treatment increased EPCT levels in *epct¯*+ pXNG4-EPCT may be due to a combination of factors including GCV’s effect wearing down and amastigotes starting rapid replication by week 14. For WT +pXNG4-EPCT amastigotes (week 14 post infection), GCV treatment had opposite effects on EPCT transcription (upregulation) and plasmid copy number (downregulation), which may reflect increased transcription from the endogenous *EPCT* locus when the episome was depleted (Fig. 5C and Fig. 6C).

4. In general the EPCT heterozygotes (+/–) had almost no phenotype in the various assays you did – indeed one copy seemed sufficient. So I was surprised that GP63 surface staining by immunofluorescence was as affected in the +/– as in the three over-expression mutants. Why do you think GP63 expression was so sensitive to EPCT haploinsufficiency?

We have provided some discussion on this issue including the potential difference between endogenous vs episomal EPCT and the type of PE being used in GPI-anchored protein synthesis (line 415-423).

5. Comment/Thought - One possibility for the effect of EPCT over-expression is that it could be exerting its effect through depletion of cellular CTP, as well as the changes you saw in PE and IPC species.

This has been included (line 385-387).

**Part III – Minor Issues: Editorial and Data Presentation Modifications**
Please use this section for editorial suggestions as well as relatively minor modifications of existing data that would enhance clarity.

Reviewer #1: I think most of fig 3 can be moved to supplementary, as can the quantification of the mRNA/copies per cell bar charts.

We have reduced the total number of main figures to nine. Fig. 3 is important to confirm the genetic manipulation and the overexpression of pXNG4-EPCT. The transcript analysis (Fig. 6) provides insight into EPCT expression beyond the DNA level.

In fig 1 .. the pathway schematic they show the 1-alkyl-2-acylPE going to -alkenyl-acyl-PE, is this really true, have theu authors knowledge of a Desaturase that works solely on the 1-alkyl-2-acylPE with no suchance of 1-alkenyl-2-acyl being the direct acceptor to the EPT?
Following on from this are the authors certain there is no 1-alkyl-2-acyl PC, hence the SAM methyl transferases only work on diacyl species?

In mammalian cells and bacteria, there are 1-O-alkyl-2-acyl-sn-glycero-3-phosphorylethanolamine desaturases which catalyze the formation of alkenyl ether bond (PMID: 4144394; PMID: 36120579; PMID: 28617934). We expect the same process in *Leishmania*.

The abundance of 1-alkyl-2-acyl PC and plasmalogen PC is quite low in *L. major* promastigotes (PMID: 33954949, PMID: 22615560). It is not proven if the PE-N-methyltransferase is specific for di-acyl PE, although most studies in the literature used di-acyl PE as the substrate (PMID: 2198947, PMID: 9370326, PMID: 25176160).

Reviewer #2: Minor comments for overall manuscript improvement:
• Figures S3 and S4: It’s unclear whether both of the heterozygotes shown were derived from the BSD gene replacement construct? The text (Lns 169-170) indicates this is the case. Did the authors try this the other way around? In other words, did they verify that the other gene replacement construct integrates appropriately into the chromosome? It’s not really a necessary control since the data with the epct-/- complemented cell line that is dependent on the presence of the episome implies that both gene copies were deleted, however, I am surprised that the authors didn’t provide Southern data showing the loss of both chromosomal alles.

Both *EPCT+/-* heterozygotes were generated with *BSD* replacement (line 450-454, supplemental Fig. S3 and Fig. S4). The loss of chromosomal *EPCT* alleles was shown in Fig. 3 (3A-3B: flanking sequence probe; 3C-3D: open reading frame probe).

• Figure S5b: I assume from the figure that just the growth point at day 2 was significantly different between the epct-/- complemented cell line and the other cell lines? What were the significance values for the other data points? I’m also curious why the cell lines appear to have different starting densities at day 0?

There was also statistically significant difference at day 3 (now added in Supplemental Fig. S6B). In these experiments, we inoculated cells at 1-2 x 10^5^ cells/ml to start the growth curve and the actual cell densities at day 0 were determined by cell counting.

• While the data in Figure 5A was highly compelling, I found the text describing this figure to be less so, mainly due to the fact that the effect of EPCT overexpression wasn’t discussed alongside the other data. I think the two sections “EPCT is indispensable for L. major amastigotes” and “EPCT overexpression leads to significantly attenuated virulence in mice” could be combined and shortened. This would serve to focus the attention on the key takeaways for this figure.

We have combined the two sections and presented them together as suggested.

• For Figure 6C, I noticed that EPCT mRNA levels were increased significantly for WT and epct-/- cell lines containing the pXNG4-EPCT episome in the presence of GCV in comparison to PBS control, which seems a little surprising. Perhaps the authors can comment a little more on this observation in the text.

This has been addressed in response to Part II, Reviewer #3, Specific comment 3.

• Figures 7-9 contain a large amount of data (changes in the level of several PE, PC, and PI metabolites for several different cell lines). I suggest moving this data to a table to make it easier to digest (though I’d focus the table on only the major differences). Figures 7 – 9 could still be included, but as supplemental data.

As described above under Part I, Reviewer #1, we have consolidated the three lipidomic figures into one (Fig. 7). Representative tandem mass spectra are now in Supplemental Fig. S9-S11.

• The starvation data is intriguing and I wonder if this phenotype might be related to defects in autophagic vesicle formation. Perhaps the authors could speculate on this possibility? Finally, I’m curious whether the authors have considered exploring the susceptibility of the complemented cell lines (WT, EPCT+/-, and epct-/-) to antileishmanial drugs? On the one had these cells seem to be more susceptible to stressors such as nutrient starvation, and pH and heat shock, but

We thank the reviewer for raising these good points. We have included autophagy in the Discussion (387-389). We have not checked the sensitivity of EPCT mutants to anti-leishmanial drugs yet in part because there are no known inhibitors or activators of EPCT which may enhance the efficacy of these drugs.

• Figure 2B and C: GFP-EPCT is clearly overexpressed in these cells, but from the western analysis it appears that there are also some likely degradation products, possibly resulting in GFP protein alone. I’m wondering how this might affect the assertion of GFP-EPCT being a cytosolic protein? The authors mention that the two enzymes immediately downstream from EPCT are localized to the ER. What is known about the enzyme immediately upstream of EPCT? Perhaps the authors can speculate on whether the cytosolic location makes sense for EPCT in the context of the other proteins?

We have addressed this issue with a new western blot (Fig. 2C). The enzymes upstream of EPCT in the Kennedy Pathway, ethanolamine kinase (EK) and choline kinase (CK), have been biochemically characterized to be bi-functional enzymes in *Leishmania* and *T. brucei* (PMID: 28754315, PMID: 18489261). In mammalian cells and yeast, EK and CK are mainly cytosolic (PMID: 16626864, PMID: 2283583, PMID: 9370318), while EPCT shows a bimodal distribution between the cytosol and smooth ER (PMID: 8195180) which is consistent with its role in PE synthesis.

• In the discussion, Lns 330-331, the phrase “it is time to recognize EPCT as the most important enzyme of the “Kennedy” pathway needs rephrasing to be less subjective.

We have removed that statement.

• In the discussion, Ln 359, the authors discuss substrate inhibition of the downstream enzymes CEPT and EPT as a plausible cause of decreased diacyl PE and PME production. I wonder what is known about the association of these different activities in other cells, specifically, whether they can be found in complex? In this context, overexpression of EPCT might also disrupt the stoichiometry of the other components of the “Kennedy” pathway, leading to decreased production of the downstream products.

That is an interesting idea although there is no evidence to support Kennedy pathway enzymes can form complexes. Perhaps EPCT overexpression increases CDP-EtN production in the cytosol but not in the ER, leading to less PME formation (line 369-377).

Reviewer #3: 6. In description of constructing the episome-assisted KOs (Lines 174-185), the text says gene replacement with BSD cassette was first, followed by episome introduction, then deletion of second allele with PAC cassette. But in Fig. 3A and using Fig. S4 scheme, it looks like PAC was first replacement then BSD. In Fig. 3A – the heterozygote shows the PAC band and the WT band (lane 2), implying it was the first gene replacement.

The error has been corrected in Supplemental Fig. S4. *BSD* was used to replace the first *EPCT* allele.

7. In Discussion, some care should be made about concluding localization of EPCT to cytoplasm using an over-expression construct (Fig. 2C). It could be the higher level of expression of EPCT could “swamp” out the localization mechanism, leading to overall cytoplasmic pattern. It’s consistent with cytoplasmic localization (and localization of other EPCT enzymes in other organisms), but localization at WT levels of expression need to be done to confirm localization.

This issue has been addressed (line 369-377).

8. In Fig. 10, it is not indicated in the Materials and Methods how the quantitation in panel C was done. Is this quantitation from a western blot or from the immunofluorescence? This info should be added to the M&M.

This issue has been addressed in response to Part II, Reviewer #2.
