# Supplementary material for: Ethanolaminephosphate cytidylyltransferase is essential for survival, lipid homeostasis and stress tolerance in Leishmania major
Source: PLoS Pathog. 2023 Jul 28;19(7):e1011112. doi: 10.1371/journal.ppat.1011112 (PMC10411802; doi:10.1371/journal.ppat.1011112)
Supplement: Attachment — Submitted filename: Response to review 07-2023.docx [file ppat.1011112.s016.docx]

We thank the editor and reviewers for their constructive feedback. We have performed the sub-cellular fractionation experiment followed by western blot as suggested (line 164-173 and 491-500). As indicated in Fig. 2D, GFP-EPCT is primarily found in the cytosolic fraction whereas the plasma membrane located LPG is mostly detected in the 10,000g pellet fraction and another cytoplasmic protein HSP83 is detected in both cytosolic and 10,000g pellet fractions.

We think Fig. 3 should be kept as a main figure because demonstrating the loss of chromosomal *EPCT* is a crucial point, and the Southern blot showed increased DNA copy number in the overexpressing cells. The journal guideline does not limit the number of figures or panels.

Response to minor edits:

*Reviewer #2: There are just a few very minor edits that I would suggest to improve the clarity of the manuscript.
1. For lns 284 and 410, where the authors indicate that the effects observed are due to a wearing down of GCV, I'd suggest changing this language to "may be due to the level of GCV likely being below the minimum effective concentration".*

We have modified the text as suggested.

*2. I believe that for ln 327 the figure the authors refer to should be 9D (not 9C) and for ln 329 Fig. 9 D-F should be Fig. 9C and F.*

We have rearranged Fig. 9C and 9D to better match the text.

*Reviewer #3: Minor edits:
Line 79 - should read "...(EtN-P) is generated from the sphingoid..."*

Corrected.

*Line 327 - This refers to Fig. 9D not 9C.*

Corrected.

*Line 329 - Refers to Fig. 9C,E,F not Fig. 9D-F*

Corrected.

*Line 340 - "combing" should be "combining"*

Corrected.

*Line 699 - Refers to Fig. 3 not Fig. 2*

Corrected.

*Line 738-739 - Statement about the structures of major PC species illustrated should be deleted. No structures are in the figure.*

Corrected. Chemical compositions for fatty acid length and saturation in PC were indicated.

*Line 742 and Fig. S11 - It looks like in the spectra that the indicated internal standard was not set at 100% relative intensity. Instead, it looks like d16:1/18:0-IPC (m/z 778.6) is set to 100% relative intensity and the other peaks normalized to that species. Maybe that IPC species was the internal standard?*

In mass spectrometry chromatogram, the most abundant peak is automatically set as 100%. In Fig. S11, the abundance of d16:1/18:0-IPC (m/z 778.6) is higher than that of the internal standard 16:0/16:0-PI (m/z 809.6). Quantitation was still done using the internal standard.

*Figure 8B - not sure what band(s) correspond(s) to LPG? Which band or set of bands is being quantified to generate the data in Fig. 8C?*

LPG is a heterologous in glycan chain length and side chain modification (PMID: 1444269). The antibody WIC79.3 detects the side chain Gal residues and a smear for LPG (PMID: 6184664). The area between 15-100 KD was used to quantify LPG.
